# An Inter-Frequency Cross-Validation Approach for Pseudo-Range Fault Detection in GNSS Relative Positioning

**DOI:** 10.3390/s25164908

**Published:** 2025-08-08

**Authors:** Zhaoyang Li, Dingjie Wang, Jie Wu

**Affiliations:** College of Aerospace Science and Engineering, National University of Defense Technology, Changsha 410073, China; lizhaoyang.nudt@alumni.nudt.edu.cn (Z.L.); wujie@nudt.edu.cn (J.W.)

**Keywords:** differential pseudorange relative positioning, fault detection and exclusion, multi-frequency multi-GNSS, cross-validation

## Abstract

For Global Navigation Satellite System (GNSS) relative positioning, faulty pseudorange measurements may lead to over-bounded relative positioning errors, which entails high-performance fault detection and exclusion (FDE). This paper proposes an effective fault detection and exclusion method for pseudorange-based GNSS relative positioning utilizing the technique of the inter-frequency cross-validation (IFCV). Multi-frequency differenced pseudorange measurements are utilized to establish multiple inter-frequency test statistics for efficient detection of multiple outliers; the conservative strategy is adopted to exclude multiple faults for robust position determination. Compared with conventional ARAIM (Advanced Receiver Autonomous Integrity Monitoring) method, the experimental results indicate that the proposed IFCV method exhibits lower false alarm rates (0.03% vs. 1.88%) and missed detection rates (0% vs. 1.02%). By artificially injecting random faults into GNSS measurements, conventional differential pseudorange-based method shows a significant decrease in positioning accuracy by 354%, while both IFCV and ARAIM methods improve positioning accuracy by 78% and 55%, respectively. Apart from advantages in accuracy over ARAIM method, the proposed IFCV demonstrates a computational efficiency improvement of 10^4^ over ARAIM.

## 1. Introduction

With the rapid development of Global Satellite Navigation Systems (GNSS), the relative positioning technology based on single-differenced pseudorange measurements between stations has been increasingly applied in diverse domains, including aircraft precision landing, vehicular positioning, maritime navigation, and geodetic surveying [1,2]. Compared to the conventional single-point positioning (SPP), the relative positioning method significantly reduces ionospheric and tropospheric errors through differencing pseudorange measurements between dual receivers, thus achieving superior positioning accuracy. Compared with carrier-phase-based precise relative positioning, this approach eliminates the need for integer ambiguity estimation and initialization filtering, consequently reducing computation complexity. However, anomalies such as satellite/receiver hardware malfunctions or multipath interference may introduce significant biases into pseudorange measurements [3]. In order to enhance the reliability of relative positioning results, it is critical to design test statistics and thresholds for pseudorange measurements accurately identifying faults and mitigating their impact [4,5].

For detecting and excluding pseudorange measurement faults, current methods can improve the positioning accuracy [6]. The conventional RAIM (Receiver Autonomous Integrity Monitoring) method constructs the test statistics and thresholds based on the least-squares (LS) principle and chi-square distribution [7,8]. When faults occur in pseudorange measurements, an alarm will be triggered. However, this method is limited to single-fault scenarios and exhibits low detection accuracy [9]. In contrast, the LS method incorporates an optimal decentralized factor to optimize test statistic via a three-dimensional critical slope. By weighting the pseudorange residuals, this approach significantly reduces the missed detection rate for satellites with large slopes and the false alarm rate for those with small slopes [10]. The correlation-weighted least squares method (CW-LSR) identifies the most likely faulty measurement through correlation analysis, then constructs the optimal test statistic based on the optimal slope. Under the condition of a maximum allowable missed detection (MD) rate, CW-LSR effectively reduces the false alarm (FA) rate [11]. A comparative study of different RAIM methods [12], including equal-weight model, elevation-angle weighting model, signal-to-noise ratio weighting model (Brunner model), and comprehensive weighting model, reveals that the classical equal-weight RAIM model leads to a higher false alarm rate. Elevation-angle weighting model and comprehensive weighting model also underperform in fault detection, while approaches based on SNR (Signal Noise Ratio) weighting model, elevation-angle weighting model, and exponential model demonstrate superior fault detection accuracy.

Based on the multi-faults assumption and the solution separation (SS) theory, the ARAIM method enables simultaneous detection of multiple faulty pseudorange measurements [13]. However, this approach necessitates the computation of multiple least-squares solutions under diverse fault assumptions, leading to a significant computational burden and occasional false alarms for normal measurements [14]. As quantified in Table 4 of our study, the FAR (false alarm rate) of ARAIM reaches 1.88%. To address these limitations, reference [15] proposes a conservative risk estimation method based on the mathematical upper bound of the horizontal positioning error (HPE) distribution. By converting the elliptic integral region into an inscribed rectangle and calculating probabilities via error function, this method dynamically adjusts horizontal protection level (HPL) candidates according to integrity risk thresholds, thereby reducing computational complexity. Reference [5] introduces a dynamic risk budget allocation strategy that continuously monitors cumulative risks during operational exposure. This approach precisely computes the upper bounds for integrity and continuity risks, which dynamically optimize the test thresholds and protection levels for SS-ARAIM. Compared to traditional Number of Effective Samples (NES) method, it significantly mitigates the conservatism in continuity risk estimation and enhances ARAIM availability, albeit at the cost of increased computational burden due to multi-dimensional Gaussian distribution calculations. For specialized applications, reference [16] develops a pseudorange fault detection and exclusion method for single faults, multiple faults, and constellation faults based on the multi-hypothesis solution separation. This approach incorporates calculations of protection level and safety envelope, providing reliable solutions for unmanned aerial vehicle (UAV) formation flights in urban environments.

As a typical application, the local area augmentation system (LAAS) employs multiple antennas at the reference station to detect faulty satellites and measurements, broadcasting integrity information to user stations [17,18]. This configuration enables the detection of pseudorange faults induced by reference station malfunctions or satellite failures. The user station adopts carrier-phase-smoothed pseudorange techniques to realize multi-solution separation (MSS) based on residual vectors, alongside with real-time dynamic protection level (PL) calculations, identifying and excluding faulty measurements. However, this scheme imposes significant computational burdens and necessitates the deployment of multiple reference stations [4].

With the advancement of multi-source data fusion technology, fault detection approaches by integrating inertial navigation systems (INS) and ultra-wideband (UWB) ranging sensors in complex environments have matured [19,20,21]. Reference [22] uses a UWB-aided fusion model based on residual sum-of-squares test to identify faulty pseudorange measurements during unmanned aerial vehicle (UAV) relative motion. The leverage-based method was employed to eliminate faulty measurements. Compared to conventional methods, this method achieves higher fault detection accuracy and lower protection levels. Reference [23] utilizes short-term position increments from INS to detect faulty pseudorange measurements. By analyzing variance distribution and integrity risk via the concept of expectation-space, this method demonstrated both lower false alarm rate and lower missed detection rate than ARAIM.

Inspired by above-mentioned methods, this paper aims to design an enhanced pseudorange gross error detection method that achieves low false alarm rate, low missed detection rate, and low computational time for multiple gross errors detection without relying on external sensor-aided information. This approach ensures that the relative positioning error in between-station differencing remains unaffected by gross error. 

This paper proposes a multi-system, multi-frequency pseudorange gross error detection and elimination approach. A novel test statistic based on IFCV is designed, whose detection accuracy is improved through least-squares adjustment. The probability distribution of the test statistic is analyzed, and the corresponding detection threshold is established. The IFCV approach leverages multi-band observations: estimated correction vector derived from multiple pseudorange observations at frequency $f_{1}$ (via LS adjustment) validate single pseudorange observation at frequency $f_{2}$, capitalizing on the superior precision of aggregated data to detect gross errors in single pseudorange observation. Utilizing the validated pseudorange measurements for relative positioning, a robust relative position solution can be realized in complex environment. Compared to conventional ARAIM method, this approach exhibits superior fault detection accuracy, reduced computation time, and enhanced relative positioning precision.

The structure of this paper is outlined as follows: Section 2.1 delineates the single-differenced (SD) pseudorange relative positioning algorithm and the least-squares estimation process, which may be influenced by pseudorange gross errors, necessitating the integration of a fault detection module. Section 2.2 describes the classical ARAIM method, serving as a benchmark for the proposed IFCV method. Section 2.3 presents the operational principles and workflow of the IFCV method proposed in this study. Section 3.1 details the airborne experimental scenario and receiver performance specifications. Section 3.2 designs a test with randomly occurring gross errors, comparing the detection efficacy and computational time of ARAIM and IFCV. Section 3.3 constructs a test involving continuously occurring minor gross errors, contrasting the detection capability of both methods and their impact on relative positioning accuracy.

## 2. Fault Detection Algorithms

### 2.1. Relative Positioning Approach with Inter-Station SD Pseudorange

Under the condition of short baseline between reference station A and user station B, measurement errors are spatially correlated between two stations. By applying SD operator to pseudorange measurements, the common errors are effectively mitigated [24]. Therefore, the inter-station SD pseudorange equation is formulated as follows:

(1) $$\Delta \rho_{AB}^{i}=\left|r^{i}(T)-r_{B}(t)\right|-\left|r^{i}(T)-r_{A}(t)\right|+c\cdot \left(\delta t_{A}-\delta t_{B}\right)+\varepsilon_{\mathrm{AB}}^{i}$$

where $\Delta \rho_{AB}^{i}=\rho_{B}^{i}-\rho_{A}^{i}$; $\rho_{A}^{i}$ and $\rho_{B}^{i}$ denote the pseudorange measurements corresponding to the ith satellite at the reference station and user station, respectively. $r^{i}(T)$ represents the position vector of the ith satellite at epoch T, while $r_{A}(t)$ and $r_{B}(t)$ denote the position vectors of the reference station and user station at epoch t. T and t represent the transmission time and reception time of the satellite signal, c denotes the light speed, $\delta t_{A}$ and $\delta t_{B}$ represent the receiver clock biases of reference station and user station, and $\varepsilon_{AB}^{i}$ indicates the SD pseudorange measurement noise.

The initial value of baseline vector is denoted as $\Delta r_{AB0}$. $\Delta r_{AB}$ and $\delta \Delta r_{AB}$ represent the true value and correction vector of the baseline vector, thus $\Delta r_{AB}=\Delta r_{AB0}+\delta \Delta r_{AB}$. The inter-station SD measurement equation is linearized as:

(2) $$\Delta \rho_{AB}^{i}-\left|r^{i}(T)-r_{A}(t)-\Delta r_{AB0}(t)\right|+\left|r^{i}(T)-r_{A}(t)\right|=-r_{B}^{i0}(T)\cdot \delta \Delta r_{AB}(t)+c\cdot \delta \Delta t_{AB}(t)+\varepsilon_{AB}^{i}$$

When the number of visible satellites is n, the equation set can be written as the following matrix form:

(3) Y=A·X+E

in which

(4) $$Y=\left[\begin{array}{c} \Delta \rho_{AB}^{1}-\left|r^{1}(T)-r_{A}(t)-\Delta r_{AB0}(t)\right|+\left|r^{1}(T)-r_{A}(t)\right|\\ \vdots \\ \Delta \rho_{AB}^{i}-\left|r^{i}(T)-r_{A}(t)-\Delta r_{AB0}(t)\right|+\left|r^{i}(T)-r_{A}(t)\right|\\ \vdots \\ \Delta \rho_{AB}^{n}-\left|r^{n}(T)-r_{A}(t)-\Delta r_{AB0}(t)\right|+\left|r^{n}(T)-r_{A}(t)\right| \end{array}\right]$$

(5) $$A=\left[\begin{array}{cc} -r_{B}^{10}(T) & 1\\ \vdots & \vdots \\ -r_{B}^{i0}(T) & 1\\ \vdots & \vdots \\ -r_{B}^{n0}(T) & 1 \end{array}\right],X=\left[\begin{array}{c} \delta \Delta r_{AB}(t)\\ c\cdot \delta \Delta t_{AB}(t) \end{array}\right],E=\left[\begin{array}{c} \varepsilon_{AB}^{1}\\ \vdots \\ \varepsilon_{AB}^{i}\\ \vdots \\ \varepsilon_{AB}^{n} \end{array}\right]$$

The unknown parameter $\hat{X}$ can be estimated with its covariance matrix $\Sigma_{\hat{X}}$ using the following equations:

(6) $$\hat{X}={\left(A^{T}\mathrm{PA}\right)}^{-1}A^{T}\mathrm{PY}$$

(7) $$\Sigma_{\hat{X}}={\left(A^{T}\mathrm{PA}\right)}^{-1}$$

where the weight matrix P is diagonal with its elements as $\frac{1}{\sigma_{i}^{2}}$, $\sigma_{i}^{2}$ denotes the reciprocal variance of the SD pseudorange noise for the ith channel. Under normal condition, $\varepsilon_{AB}^{i}$ and $\varepsilon_{\hat{X}}$ follow normal distribution with zero expectation.

### 2.2. Fault Detection Method Based on ARAIM

The ARAIM method represents a conventional approach for pseudorange fault detection, based on the solution separation hypothesis within the positioning domain [25]. This methodology employs multiple-fault hypotheses with corresponding position deviations to identify faulty pseudorange measurements [26]. The definition of the hypotheses is presented as follows:

**Hypothesis** **H_0_.** *There is no fault in the measurements.*

**Hypothesis** **H_*i*_.** *There are faults in the measurements of* i *th subset.*

The projection matrix from the measurement domain to the positioning domain is defined as

(8) $$S={\left(A^{T}\mathrm{PA}\right)}^{-1}A^{T}P$$

Corresponding to the fault hypothesis, the position solution vector is expressed as

(9) $${\hat{x}}_{i}=S_{i}\times Y$$

where $S_{i}$ represents the projection matrix corresponding to the Hypothesis $H_{i}$.

The test statistics of ARAIM is defined as follows

(10) $$\left\{\begin{array}{c} T_{i,N}={\hat{x}}_{i,N}-{\hat{x}}_{0,N}\\ T_{i,E}={\hat{x}}_{i,E}-{\hat{x}}_{0,E}\\ T_{i,D}={\hat{x}}_{i,D}-{\hat{x}}_{0,D} \end{array}\right.$$

where $T_{i,N}$, $T_{i,E}$ and $T_{i,D}$ represent test statistics for the northern, eastern, and vertical directions corresponding to the ith fault hypothesis $H_{i}$; ${\hat{x}}_{i,N}$, ${\hat{x}}_{i,E}$, and ${\hat{x}}_{i,D}$ represent the estimated value of the three-dimensional positioning solution corresponding to the ith fault hypothesis; ${\hat{x}}_{0,N}$, ${\hat{x}}_{0,E}$ and ${\hat{x}}_{0,D}$ represent the estimated value of the three-dimensional position solution corresponding to the fault-free hypothesis.

In the absence of pseudorange faults, all test statistics should follow the zero-mean normal distribution, i.e.,

(11) $$\left\{\begin{array}{c} T_{i,N}∼N(0,\sigma_{i,N}^{2})\\ T_{i,E}∼N(0,\sigma_{i,E}^{2})\\ T_{i,D}∼N(0,\sigma_{i,D}^{2}) \end{array}\right.$$

where $\sigma_{i,N}^{2}$, $\sigma_{i,E}^{2}$, and $\sigma_{i,D}^{2}$ represent the three diagonal elements of the test statistics covariance matrix corresponding to the fault hypothesis $H_{i}$.

If faults occur in a subset of pseudorange measurements, the expectation of the test statistic will significantly deviate from zero. Consequently, the test statistic exceeds the predetermined threshold with high probability, thereby triggering user alerts. The threshold is determined as follows:

(12) $$\left\{\begin{array}{c} TH_{i,N}=-\Phi^{-1}(\frac{P_{FA}}{4N_{\mathrm{hypo}}})\cdot \sigma_{i,N}\\ TH_{i,E}=-\Phi^{-1}(\frac{P_{FA}}{4N_{\mathrm{hypo}}})\cdot \sigma_{i,E}\\ TH_{i,D}=-\Phi^{-1}(\frac{P_{FA}}{2N_{\mathrm{hypo}}})\cdot \sigma_{i,D} \end{array}\right.$$

where $TH_{i,N}$, $TH_{i,E}$ and $TH_{i,D}$ represent the test thresholds for the north, east, and down directions under the fault hypothesis $H_{i}$; $P_{FA}$ is the given false alarm rate, and $N_{\mathrm{hypo}}$ is the total number of fault hypotheses; $\Phi^{-1}(x)$ denotes the quantile function of the standard normal distribution, with the definition of Φ(x) as follows:

(13) $$\Phi (x)=\int_{-\infty }^{x}\frac{1}{\sqrt{2\pi }}\cdot e^{-\frac{\tau^{2}}{2}}d\tau$$

### 2.3. The Proposed Fault Detection Method Based on IFCV

This paper proposes an IFCV-based method to detect faults in SD pseudorange measurements. Least-squares estimation is performed on the SD pseudorange measurements across multiple channels of a single frequency ($f_{1}$) to estimate corrections ($\delta r_{AB,f_{1}}$) to the inter-station baseline vector. Subsequently, SD pseudorange measurements for another frequency ($f_{2}$) are predicted channel-by-channel. A new test statistic is constructed by comparing these predicted values with the actual SD pseudorange measurements. If the test statistic exceeds predefined threshold, the corresponding channel’s pseudorange is identified as contaminated by fault.

Utilizing all pseudorange measurements at frequency $f_{1}$, the unknown parameters vector ${\hat{X}}_{f_{1}}=\left[\begin{array}{cc} \delta \Delta r_{AB,f_{1}}(t) & c\cdot \delta \Delta t_{AB,f_{1}}(t) \end{array}\right]$ is estimated with LS method, as shown in Equation (6). ${\hat{X}}_{f_{1}}$ is employed to predict the pseudorange measurements $\Delta {\tilde{\rho }}_{AB,f_{2}}^{i}$ for each channel on frequency $f_{2}$, i.e., (taking ith satellite as an example, where i=1,…,n).

(14) $$\Delta {\tilde{\rho }}_{AB,f_{2}}^{i}=\left|r^{i}(T)-r_{A}(t)-r_{AB0}(t)\right|-\left|r^{i}(T)-r_{A}(t)\right|-r_{B}^{i0}(T)\cdot \delta \Delta r_{AB,f_{1}}(t)+c\cdot \delta \Delta t_{AB,f_{1}}$$

Since the estimation error $\varepsilon_{{\hat{X}}_{f_{1}}}$ follows a normal distribution with zero expectation, the prediction error $\varepsilon_{\Delta {\tilde{\rho }}_{AB,f_{2}}^{i}}$ after linear projection also conforms to a zero-mean normal distribution.

Using the covariance propagation law, the variance of the predicted value $\Delta {\tilde{\rho }}_{AB,f_{2}}^{i}$ is derived as follows:

(15) $$\sigma_{\Delta {\tilde{\rho }}_{AB,f_{2}}^{i}}^{2}=A_{i}\cdot \Sigma_{{\hat{X}}_{f_{1}}}\cdot A_{i}^{T}$$

where $A_{i}={\left[\begin{array}{cc} -r_{B}^{i0}(T) & 1 \end{array}\right]}^{T}$ is the coefficient matrix of the measurement equation for *i*th satellite; $\Sigma_{{\hat{X}}_{f_{1}}}$ is the variance matrix of the estimated parameters ${\hat{X}}_{f_{1}}$, deriving from the conservative variance of SD pseudorange measurements at frequency $f_{1}$, as shown in Equation (7).

Due to the adjustment effect of least-squares estimation, the predicted value $\Delta {\tilde{\rho }}_{AB,f_{2}}^{i}$ exhibits higher precision and robustness than individual actual observations $\Delta \rho_{AB,f_{2}}^{i}$. This enables gross error detection by comparing $\Delta \rho_{AB,f_{2}}^{i}$ with $\Delta {\tilde{\rho }}_{AB,f_{2}}^{i}$. Under normal conditions, $\left|\Delta \rho_{AB,f_{2}}^{i}-\Delta {\tilde{\rho }}_{AB,f_{2}}^{i}\right|$ converges near zero. When $\Delta \rho_{AB,f_{2}}^{i}$ contains gross errors, a significant deviation from $\Delta {\tilde{\rho }}_{AB,f_{2}}^{i}$ occurs. The proposed IFCV method operates on this principle to detect and eliminate pseudorange gross errors. The computation methods for test threshold and test statistic are quantitatively analyzed as follows:

The test statistic is constructed from the residual between the actual and predicted SD pseudorange measurements at frequency $f_{2}$:

(16) $$T_{f_{2}}^{i}=\Delta \rho_{AB,f_{2}}^{i}-\Delta {\tilde{\rho }}_{AB,f_{2}}^{i}$$

where $\Delta \rho_{AB,f_{2}}^{i}$ denotes the SD pseudorange measurement of *i*th satellite at frequency $f_{2}$.

Under the null hypothesis that the SD pseudorange measurement $\Delta \rho_{AB,f_{2}}^{i}$ at frequency $f_{2}$ is free of gross error, the errors ($\varepsilon_{\Delta \rho_{AB,f_{2}}^{i}}$ and $\varepsilon_{\Delta {\tilde{\rho }}_{AB,f_{2}}^{i}}$) follow zero-mean normal distributions. Consequently, the test statistic $T_{f_{2}}^{i}$ follows a zero-mean normal distribution. Conversely, if gross error exists in the SD pseudorange measurement $\Delta \rho_{AB,f_{2}}^{i}$, $T_{f_{2}}^{i}$ will follow a non-central normal distribution.

(17) $$\left\{\begin{array}{c} H_{0}:T_{f_{2}}^{i}∼N(0,\sigma_{T}^{2})\\ H_{1}:T_{f_{2}}^{i}∼N(\mu ,\sigma_{T}^{2}),\mu \neq 0 \end{array}\right.$$

where $\sigma_{T}^{2}$ denotes the standard deviation of $T_{f_{2}}^{i}$, i.e., $\sigma_{\Delta \rho_{AB,f_{2}}^{i}}^{2}$ represents the normal error variance.

(18) $$\sigma_{T}^{2}=\sigma_{\Delta \rho_{AB,f_{2}}^{i}}^{2}+\sigma_{\Delta {\tilde{\rho }}_{AB,f_{2}}^{i}}^{2}$$

Given a significance level α, the test threshold TH is calculated as the following:

(19) $$TH_{f_{2}}^{i}=-\Phi^{-1}(\frac{\alpha }{2})\cdot \sigma_{T}$$

where $\Phi^{-1}(x)$ is the inverse cumulative distribution function (CDF) of the standard normal distribution.

When the test statistic $T_{f_{2}}^{i}$ is greater than the threshold $TH_{f_{2}}^{i}$, it indicates that the pseudorange observation is free of gross errors and can be utilized for positioning. Conversely, if the test statistic $T_{f_{2}}^{i}$ exceeds the threshold $TH_{f_{2}}^{i}$, it indicates that there is a gross error in the pseudorange observation and needs to be rejected. The procedure describes the gross error detection for the observations at frequency $f_{2}$. A parallel detection process is required for the observations at frequency $f_{1}$, with both frequencies processed simultaneously. After pseudorange observations from all channels are validated by the IFCV method, the relative positioning solution is computed using dual-frequency ionosphere-free combination and between-station differencing techniques, then delivered to end-users.

**Figure 1:**
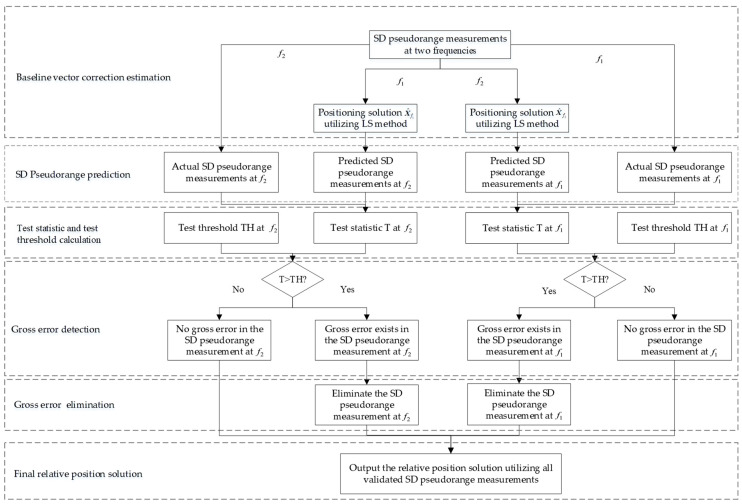
The flowchart of the proposed IFCV-based method.

The algorithmic workflow for the proposed IFCV-based method is detailed below, as illustrated in Figure 1.

(1) Baseline vector correction estimation: Apply LS method to SD pseudorange measurements $\Delta \rho_{AB\;,\;f_{1}}^{i}$ across multiple channels at frequency $f_{1}$ to estimate the baseline vector correction $\delta \Delta r_{AB}(t)$ and receiver clock bias $\delta \Delta t_{AB,f_{1}}(t)$. Residual vector screening is applied after Equation (6), where pseudorange observations corresponding to out-of-tolerance residual elements are eliminated, ensuring the accuracy and robustness of the state parameters $\delta \Delta r_{AB}(t)$ and $\delta \Delta t_{AB,f_{1}}(t)$.
(2) Pseudorange prediction: Predict the SD pseudorange $\Delta {\tilde{\rho }}_{AB\;,\;f_{2}}^{i}$ at frequency $f_{2}$ utilizing the geometric coefficient matrix A and the updated baseline vector $\delta \Delta r_{AB}(t)$.
(3) Test statistic calculation: Compute the test statistic $T_{f_{2}}^{i}$ with the residual between predicted and actual pseudorange observations.
(4) Gross error detection: Calculate the test threshold $TH_{f_{2}}^{i}$ based on the measurement variance $\sigma_{T}^{2}$. Channels where $T_{f_{2}}^{i}>TH_{f_{2}}^{i}$ are flagged and eliminated.
(5) Channel-by-channel detection: Repeat Steps 2–4 to exclude faulty pseudorange observations for all channels at frequency $f_{2}$.
(6) Frequency-by-frequency detection: Repeat Steps 1–5 to exclude faulty pseudorange observations for all channels at frequency $f_{1}$.
(7) Relative position solution: Perform relative positioning between-station differencing techniques using validated measurements, and output the final relative position vector $r_{\mathrm{AB}}$.

## 3. Experimental Validation

### 3.1. Field Test Setup

To evaluate the comparative performance of different FDE methods (i.e., IFCV and ARAIM), a flight test was conducted at a general aviation airport in Inner Mongolia using a fixed-wing aircraft. Throughout the flight, a static ground reference station is established to allow for differential pseudorange positioning. Stochastic pseudorange faults (10–20 m) were artificially injected into selected satellite channels (C1 in BDS). Both methods will be evaluated using the faulty dataset for comparison. Performance metrics include relative positioning error, false alarm rate, missed detection rate (MDR), and computational efficiency.

**Figure 2:**
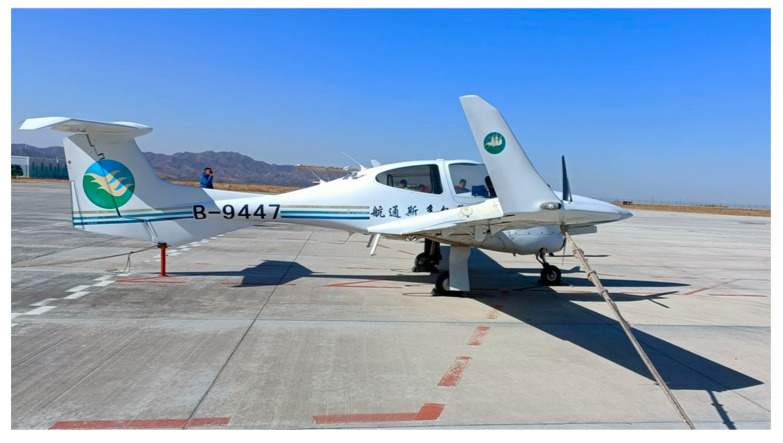
GNSS antenna installation position.

**Figure 3:**
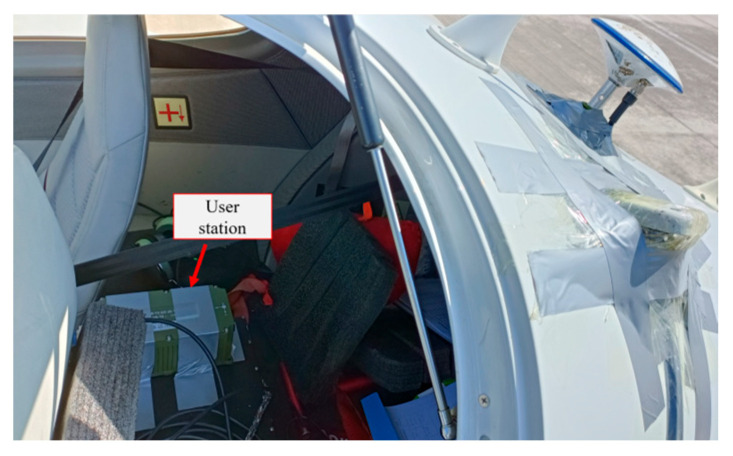
Layout of user station receiver.

The test employed a 4-seat aircraft with an equipment bay in the rear cabin. A GNSS receiver was mounted on the rear seat, while its antenna was installed externally on the dorsal fuselage, as shown in Figure 2. The installation layout of the user station receiver is shown in Figure 3.

**Figure 4:**
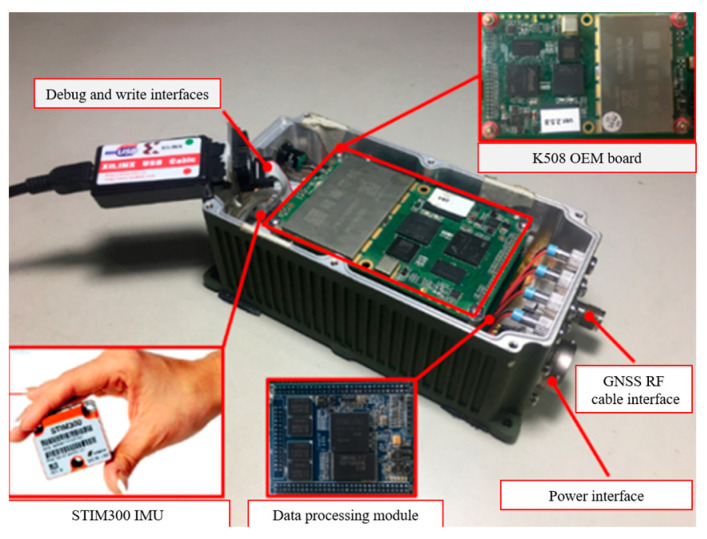
Internal configuration of navigation receiver.

The GNSS receiver is a self-developed multi-constellation GNSS/INS integrated prototype system, which is comprised of a data processing board and a K508 OEM (Original Equipment Manufacturer) board, as shown in Figure 4. Product specifications are provided in Table 1 [27]. The K508 OEM board is developed and manufactured by ComNav Technology Ltd in Shanghai, China.

**Figure 5:**
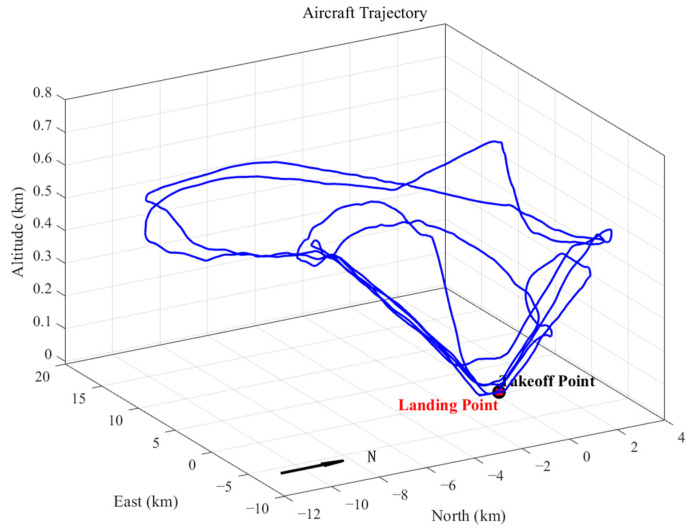
Three-dimensional flight trajectory of fixed-wing aircraft.

During the experiment, the fixed-wing aircraft completed a flight duration of approximately 1 h, with the following key trajectory parameters recorded relative to the ground reference station, as shown in Figure 5:

- Maximum baseline distance: 17.3 km
- Peak altitude: 745 m (ellipsoidal height)
- Flight pattern: Four closed-circuit trajectories

**Figure 6:**
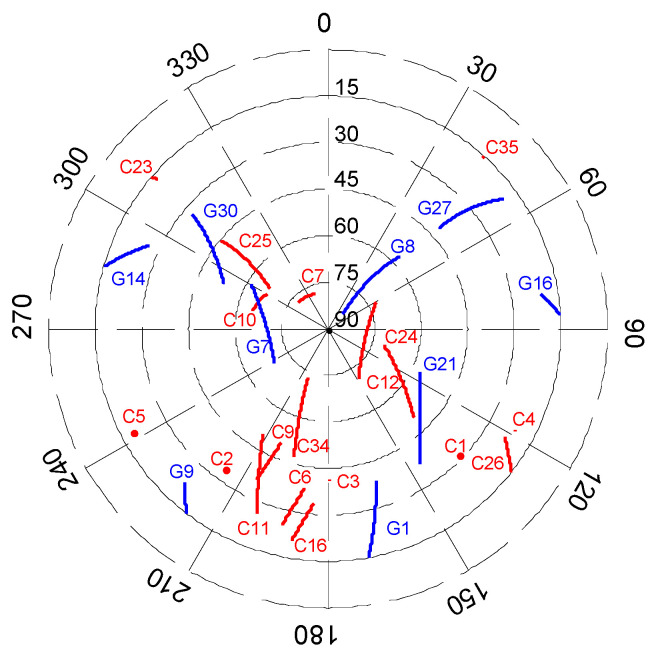
Zenithal plane projection of visible satellite trajectories (i.e., G1 denotes the first satellite in GPS, and C1 denotes the first satellite in BDS).

The ground reference station was deployed nearby the airport runway. Throughout the experiment’s duration, the number of visible satellites remained stable ranging from 23 to 26. The motion trajectory projected in the zenithal plane relative to the static reference station is illustrated in Figure 6.

### 3.2. Random Gross Error Detection Test

Four schemes are implemented for post-processing test, labeled as “scheme A”, “scheme B”, “scheme C”, and “scheme D”. Their descriptions are listed as follows:

- Scheme A: Ordinary pseudorange-based relative positioning using the observations without artificial gross errors.
- Scheme B: Pseudorange-based relative positioning using the observations contaminated by artificial gross errors in C1 satellite without detection.
- Scheme C: ARAIM-based differential pseudorange-based positioning with artificial gross errors.
- Scheme D: The proposed IFCV-based differential pseudorange-based positioning artificial gross errors.

**Figure 7:**
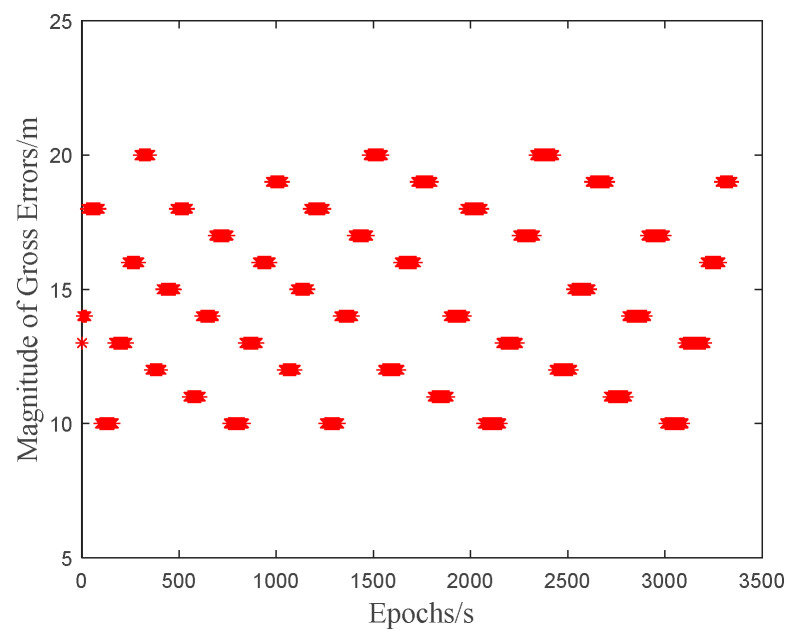
Magnitude of added random gross errors.

During the test, gross errors are randomly added into the pseudorange observations on both frequencies (B1/B3) of the C1 satellite at each epoch, as illustrated in Figure 7. The experimental parameters are presented in Table 2.

**Figure 8:**
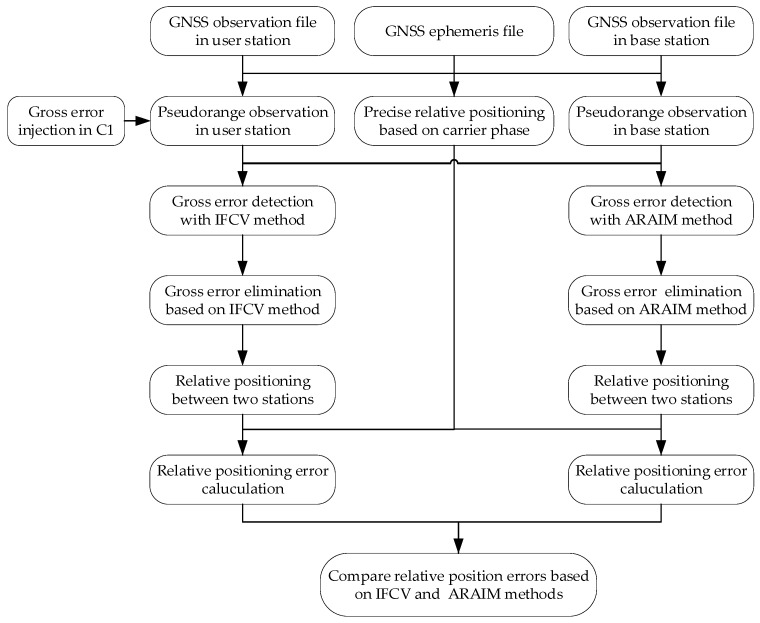
The experimental test flow of gross error elimination.

Figure 8 illustrates the experimental test flow of gross error elimination and differenced pseudorange-based relative positioning.

The effectiveness of the proposed IFCV-based method is verified from the following three aspects.

#### 3.2.1. Relative Positioning Accuracy Analysis

**Figure 9:**
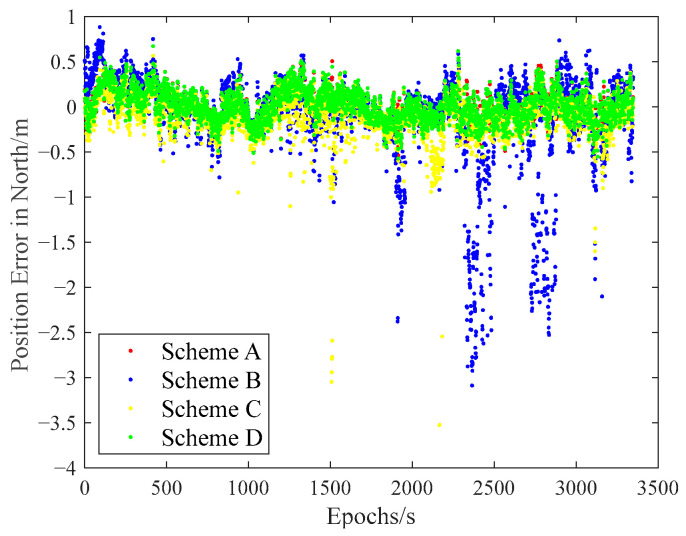
Relative position error in northern direction with random gross errors.

**Figure 10:**
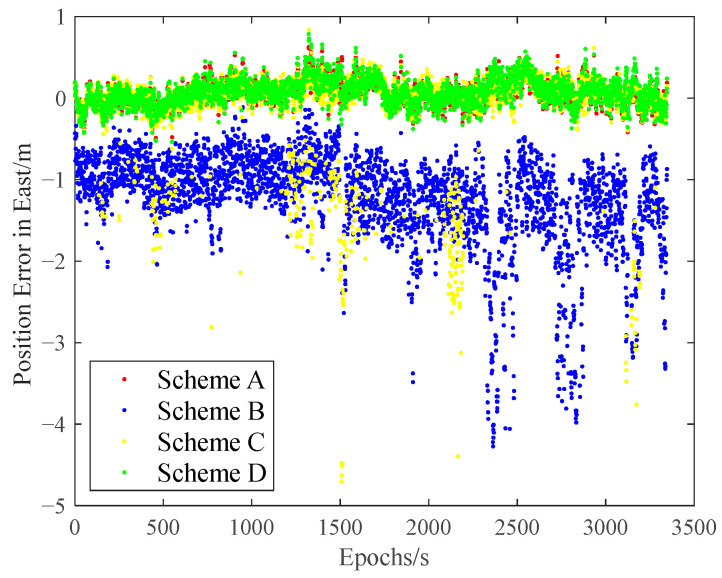
Relative position error in eastern direction with random gross errors.

**Figure 11:**
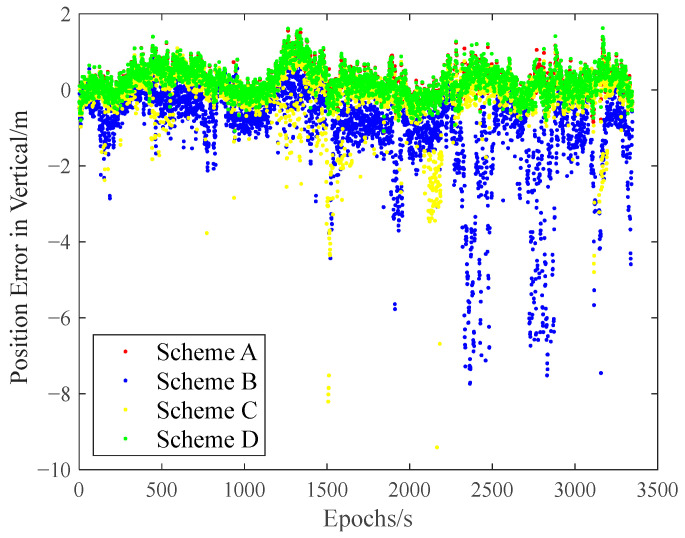
Relative positioning error in vertical direction with random gross errors.

The carrier-phase-based precise relative positioning results are adopted as the reference baseline vectors. The relative position errors are computed in the north, east, and vertical directions with their comparisons illustrated in Figure 9, Figure 10 and Figure 11.

Initial analysis indicates that Scheme A and Scheme D demonstrate similar relative positioning errors, whereas Scheme C exhibits significantly larger errors. Scheme B displays the most pronounced positioning deviations. These findings demonstrate that both the IFCV and ARAIM methods effectively detect and eliminate pseudorange faults. Furthermore, the proposed IFCV method outperforms ARAIM in error detection efficacy.

To quantitatively compare the performance divergence between these two methods, the relative positioning errors of all four schemes are statistically summarized across three orthogonal directions (North, East, Vertical) in Table 3.

As indicated in Table 3, the introduction of artificial faults into pseudorange measurements increases positioning errors by 354%, compared to fault-free conditions. However, both ARAIM and IFCV methods significantly reduce relative positioning errors by 55% and 78%, respectively.

#### 3.2.2. Fault Detection Efficiency Analysis

To quantitatively evaluate the fault detection capabilities for both ARAIM and IFCV, the false alarm rate (FAR) is assessed under fault-free conditions (Scheme A), while the missed detection rate (MDR) is assessed after introducing artificial faults (Schemes C and D). The statistical results are presented in Table 4.

As indicated in Table 4, FAR and MDR of the IFCV method are significantly lower than those of ARAIM. This finding explains the reduced relative positioning error of Scheme D compared to Scheme C in Table 3.

#### 3.2.3. Computational Efficiency Analysis

**Figure 12:**
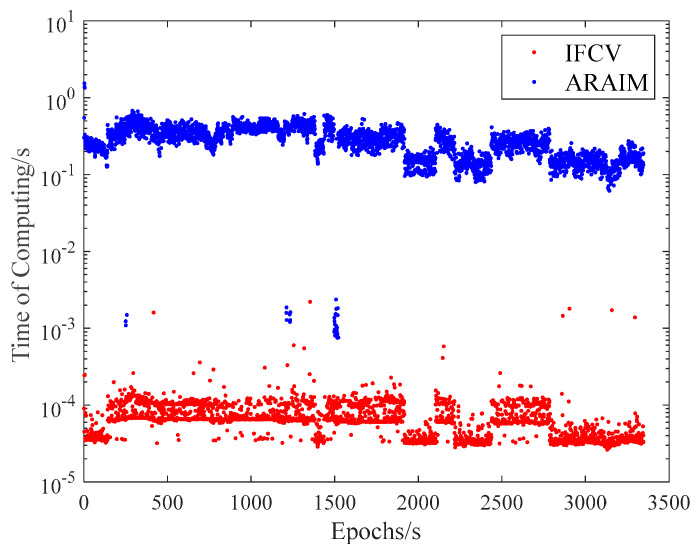
Computational time of ARAIM and IFCV methods.

To quantitatively compare the computational efficiency of ARAIM and IFCV, the processing time is recorded for each method. The results are presented in Figure 12 and Table 5. The computational tasks are performed on a Legion Y9000P IRX8 laptop with the following hardware configuration, which is made by Lenovo Group Ltd in Hefei, China:

Processor: 13th Gen Intel^®^ Core™ i9-13900HX (24 cores, base frequency 2.2 GHz);

Memory: 16GB SK Hynix DDR5 5600MHz dual-channel RAM;

Graphics: NVIDIA GeForce RTX™ 4060 Laptop GPU.

Both Figure 12 and Table 5 demonstrate that the computational time of ARAIM significantly exceeds that of IFCV. This divergence arises from their operational principles: ARAIM requires high-dimensional matrix inversion for each fault subset. As the number of visible satellites increases, the quantity of fault subsets and matrix dimensions grows substantially, leading to exponential computational demands. As a contrast, the IFCV method requires only three matrix inversions throughout its workflow. As a result, its computational time is decreased by approximately 99.976% lower than ARAIM.

### 3.3. Consecutive Small Gross Error Detection Test

To comparatively evaluate the capability of ARAIM and IFCV methods in detecting consecutive small gross errors, four experimental schemes are designed:

- Scheme A: Ordinary pseudorange-based relative positioning using the observations without artificial gross errors.
- Scheme B: Small gross errors (4–8 m) were continuously injected into two pseudorange observations (B1 and B3) of the C1 satellite, but no detection method is performed (the normal pseudorange measurement error for BDS satellites is characterized by a Signal-in-Space Ranging Error (SISRE) of 4.6 m at 95% confidence level [28]).
- Scheme C: Based on Scheme B’s setup, ARAIM method is applied to detect and eliminate gross errors.
- Scheme D: Based on Scheme B’s setup, the proposed IFCV method is applied to detect and eliminate gross errors.

**Figure 13:**
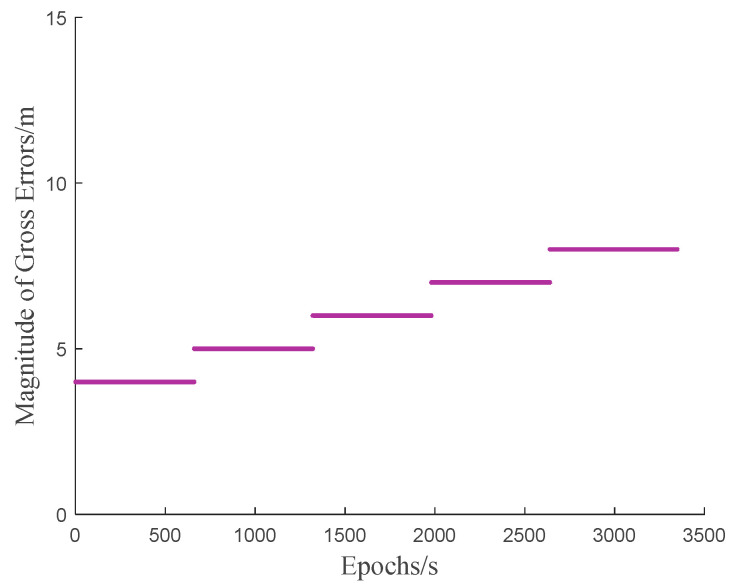
Magnitude of added consecutive small gross errors (4~8 m).

The magnitude and distribution epochs of injected gross errors are illustrated in Figure 13.

**Figure 14:**
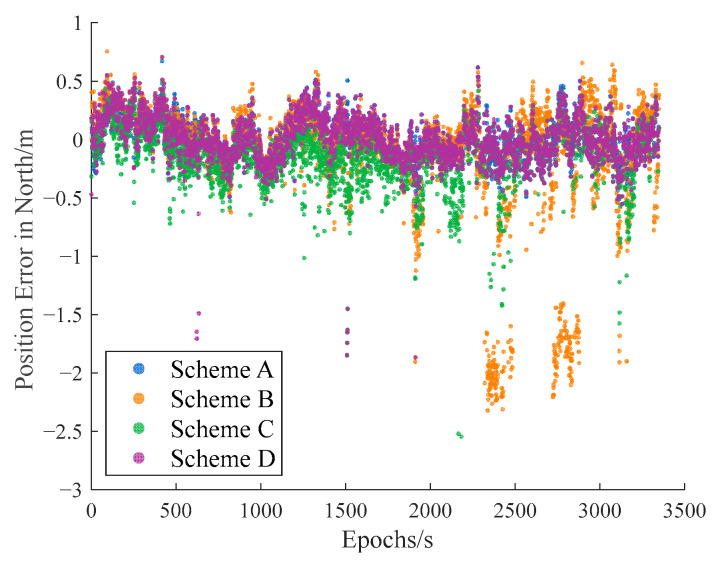
Relative position error in northern direction with consecutive small gross errors.

**Figure 15:**
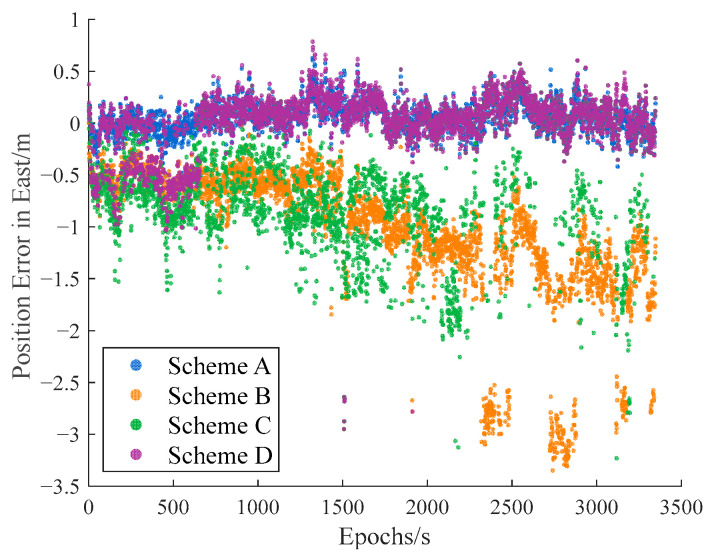
Relative position error in eastern direction with consecutive small gross errors.

**Figure 16:**
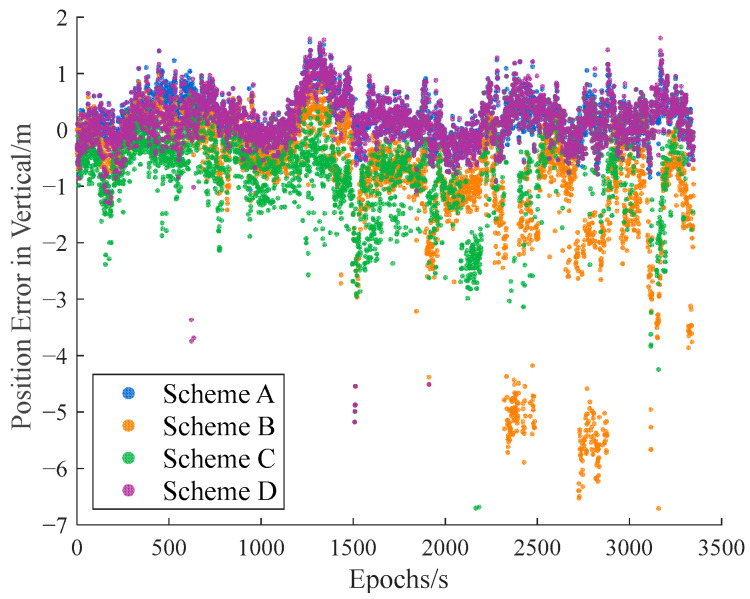
Relative position error in vertical direction with consecutive small gross errors.

Carrier-phase-based precise relative positioning solutions serve as the truth value for baseline vector. The effectiveness of IFCV is validated by comparing relative positioning errors and missed detection rate (Figure 14, Figure 15 and Figure 16).

As shown in Table 6, Scheme B exhibits a threefold increase in relative positioning error compared to Scheme A, confirming that gross errors significantly degrade positioning accuracy. Scheme C (ARAIM) reduces positioning errors by 40% relative to Scheme B, indicating partial mitigation of gross error impacts. Scheme D (IFCV) further reduces errors by 51% relative to Scheme C, with positioning errors marginally exceeding Scheme A’s nominal level.

To quantify detection accuracy for consecutive small gross errors, missed detection rates of ARAIM and IFCV are statistically analyzed (Scheme C vs. D) in Table 7.

As shown in Table 7, IFCV method achieves missed detection rate of only 12.31%, corresponding to 87.68% successful detection rate of consecutive small gross errors. This missed detection rate represents only 32.7% of ARAIM Most undetected errors occur during 4m gross error injections, where impacts on relative positioning are negligible.

## 4. Conclusions

This paper proposes an IFCV method for detecting and eliminating pseudorange gross errors. Validated through airborne flight experiments, the IFCV method demonstrates significant improvements over the ARAIM method. For random large pseudorange gross errors (10–20 m), the IFCV method reduced the MDR from 1.02% to 0%, decreased the relative positioning error from 1.02 m to 0.51 m, and reduced the average computational time from 0.28 s to 0.069 milliseconds. For constant small gross errors (4–8 m), compared to ARAIM, IFCV lowered the pseudorange MDR from 37.66% to 12.31% and decreased the relative positioning error from 1.24 m to 0.61 m. The superior performance of the IFCV method stems from several factors: (1) The high-precision baseline vector is calculated with multiple single-frequency pseudorange observations and LS estimation, and then gross errors detection is performed individually on another frequency, thereby enhancing detection accuracy. (2) The lower MDR of IFCV effectively mitigates the impact of gross errors on relative positioning error, leading to significantly improved positioning accuracy. (3) IFCV requires only two least squares solutions and matrix inversion operations, resulting in a significant reduction in computational time. In contrast, the ARAIM method performs numerous matrix inversions as it calculates test statistics within the position domain for multiple fault hypotheses, consuming considerable computational resources. Furthermore, when multiple pseudorange observations simultaneously exhibit small gross errors, ARAIM’s limited number of fault hypotheses leads to a significant increase in MDR. The IFCV method is free from these limitations.

Primarily designed to detect pseudorange gross errors in relative positioning, the IFCV method offers advantages including a low MDR, high positioning accuracy, and low computational time. These attributes make it suitable for applications such as aircraft landing, UAV formation flight, and vehicle–road collaborative navigation, where it enhances the robustness of relative positioning results and demonstrates broad application prospects. Moreover, the method enables independent detection of different pseudorange observations, eliminating post-test error correlations between channels, which is beneficial for analyzing the integrity risk of relative positioning results. Our future work will focus on three key areas: (1) Evaluating the algorithm’s performance under complex scenarios like urban canyons and strong electromagnetic interference; (2) Investigating tight or deep coupling of the IFCV method with other multi-source sensors (e.g., INS, LiDAR, visual navigation) to further enhance detection accuracy; and (3) Extending the method’s application to cycle slip detection for carrier phase observations, improving the robustness and integrity of precise relative positioning algorithms.

## Figures and Tables

**Table 1:** Product specifications of K508 OEM board.

Parameter Type	System	Frequency Band	Accuracy
Pseudorange Measurement Accuracy	GPS	L1	10 cm
		L2	10 cm
	BDS	B1	10 cm
		B2	10cm
		B3	5 cm
Carrier Phase Measurement Accuracy	GPS	L1	1 mm
		L2	1 mm
	BDS	B1	1 mm
		B2	1 mm
		B3	1 mm
Timing Accuracy	All	—	20 ns
Standard Point Positioning Accuracy	Single-frequency	Horizontal	3.0 m (1σ)
		Vertical	5.0 m (1σ)
	Dual-frequency	Horizontal	1.5 m (1σ)
		Vertical	3.0 m (1σ)

Notes: 1. All accuracy values are given under conditions of PDOP ≤ 4 and open-sky environments. 2. “—” indicates no data provided for the specified frequency band. 3. 1σ denotes one standard deviation, representing 68% confidence level.

**Table 2:** Experimental parameters for relative positioning with fault detection.

Sampling Rate	1 Hz
Significance level	0.01
RMS of Pseudorange Measurement Noise	2 m
Range of Random Added Fault Errors	10~20 m
Faulty Probability of Single Satellite	1 × 10^−5^

**Table 3:** Relative positioning error comparison among 4 schemes with random gross errors.

Scheme	North/m	East/m	Vertical/m	Total/cm
A	0.17	0.16	0.44	0.50
B	0.56	1.39	1.70	2.27
C	0.25	0.58	0.80	1.02
D	0.17	0.17	0.45	0.51

**Table 4:** Detection efficiency comparison between ARAIM and IFCV methods.

Method	Total Number of Epochs	Number of False Alarm Epochs	False Alarm Rate	Number of Missed Detection Epochs	Missed Detection Rate
ARAIM	3347	63	1.88%	34	1.02%
IFCV		1	0.03%	0	0

**Table 5:** Comparison of computational time between ARAIM and IFCV methods.

Method	Total Number of Epochs	Max Time of Computing/s	Min Time of Computing/s	Mean Time of Computing/s
ARAIM	3347	1.54	7.50 × 10^−4^	0.28
IFCV		2.21 × 10^−3^	2.63 × 10^−5^	6.86 × 10^−5^

**Table 6:** Relative positioning error comparison among 4 schemes with consecutive small gross errors.

Scheme	North/m	East/m	Vertical/m	Total/m
A	0.17	0.16	0.44	0.50
B	0.52	1.22	1.60	2.08
C	0.27	0.78	0.93	1.24
D	0.20	0.29	0.50	0.61

**Table 7:** Missed detection rate Comparison between ARAIM and IFCV methods.

Method	Total Number of Epochs	Number of Successful Detection Epochs	Successful Detection Rate	Number of Missed Detection Epochs	Missed Detection Rate
ARAIM	3347	2086	62.34%	1261	37.66%
IFCV		2935	87.69%	412	12.31%

## Data Availability

The raw data supporting the conclusions of this article will be made available by the authors on request.

## Abbreviations

The following abbreviations are used in this manuscript:

ARAIM	Advanced receiver autonomous integrity monitoring
CW-LSR	Correlation-weighted least squares method
FAR	False alarm rate
FDE	Fault detection and exclusion
GNSS	Global navigation satellite system
HPE	Horizontal positioning error
HPL	Horizontal protection level
IFCV	Inter-frequency cross-validation
INS	Inertial navigation system
LAAS	Local area augmentation system
LS	Least-squares
MD	Missed detection
MDR	Missed detection rate
MSS	Multi-solution separation
NES	Number of effective samples
OEM	Original equipment manufacturer
PL	Protection level
RAIM	Receiver autonomous integrity monitoring
SD	Single-differenced
SNR	Signal noise ratio
SPP	Single-point positioning
SS	Solution separation
UAV	Unmanned aerial vehicle
UWB	Ultra-wideband

## References

[1] Li Q., Dong Y., Wang D., Wu J., Zhang L. (2022). Real-Time Precise DGNSS/INS Integrated Relative Positioning with High Output Rate and Low Broadcast Rate for Kinematic-to-Kinematic Applications. Remote Sens..

[2] Dong Y., Zhang L., Wang D., Li Q., Wu M. (2020). Lowlatency, highrate, highprecision relative positioning with moving base in real time. GPS Solut..

[3] Chen H., Sun R., Cheng Q., Yang L. (2023). A factor set-based GNSS fault detection and exclusion for vehicle navigation in urban environments. GPS Solut..

[4] Pullen S., Luo M., Gleason S., Xie G., Lee J., Akos D., Enge P., Pervan B. GBAS Validation Methodology and Test Results from the Stanford LAAS Integrity Monitor Testbed. Proceedings of the 13th International Technical Meeting of the Satellite Division of The Institute of Navigation.

[5] Du J., Wang H., Fang K., Wang Z., Zhu Y. (2025). Continuity and integrity allocation over operational exposure time for ARAIM fault detection algorithm. Chin. J. Aeronaut..

[6] Li R., Li L., Li M., Cheng L., Wang L., Zhang J. (2025). Integrity-directed fault exclusion based on maximum a posteriori probability for multi-constellation advanced RAIM. GPS Solut..

[7] Blanch J., Walker T., Enge P., Lee Y., Pervan B., Rippl M., Spletter A., Kropp V. (2015). Baseline advanced RAIM user algorithm and possible improvements. IEEE Trans. Aerosp. Electron. Syst..

[8] Ma X., Yu K., Wang E., He X., Lu T., Li Q. (2021). Evaluation of BDS and GPS RAIM availability based on data collected in June 2020. Geod. Geodyn..

[9] Cheng Q., Chen P., Sun R., Wang J., Mao Y., Ochieng W.Y. (2021). A New Faulty GNSS Measurement Detection and Exclusion Algorithm for Urban Vehicle Positioning. Remote Sens..

[10] Sun G., Xu C., Song D., Jian Y. (2020). An enhanced least squares residual RAIM algorithm based on optimal decentralized factor. Chin. J. Aeronaut..

[11] Song D., Shi C., Wang Z., Wang C., Jing G. (2020). Correlation-weighted least squares residual algorithm for RAIM. Chin. J. Aeronaut..

[12] Wang C., Yang C., Shi J. (2025). Impact of different weighting methods on GNSS receiver integrity fault detection. J. Navig. Position.

[13] Zhai Y., Joerger M., Pervan B. Bounding Continuity Risk in H-ARAIM FDE. Proceedings of the ION Pacific PNT Conference.

[14] Zhang Y., Wang L., Fan L. (2019). Study on MHSS ARAIM algorithm combined with gross error detection. Acta Geod. Cartogr. Sin..

[15] Liu B., Gao Y., Gao Y., Wang S. (2022). HPL calculation improvement for Chi-squared residual-based ARAIM. GPS Solut..

[16] Wang S., Zhan X., Zhai Y., Chi C., Shen J. (2021). Highly reliable relative navigation for multi-UAV formation flight in urban environments. Chin. J. Aeronaut..

[17] Lee J. LAAS Position Domain Monitor Analysis and Test Results for CAT II/III Operations. Proceedings of the the 17th International Technical Meeting of the Satellite Division of The Institute of Navigation (ION GNSS 2004).

[18] Wen H., Havlicek J., Fagan J. B-value research for FAA LAAS station integrity and fault detection. Proceedings of the 2004 National Technical Meeting of The Institute of Navigation.

[19] Wu Y. (2014). Autonomous Integrity Monitoring of Tightly Coupled GNSS/INS Navigation System. Acta Geod. Cartogr. Sin..

[20] Alghananim M.S., Feng C., Feng Y., Ochieng W.Y. (2025). Machine Learning-Based Fault Detection and Exclusion for Global Navigation Satellite System Pseudorange in the Measurement Domain. Sensors.

[21] Bhattacharyya S. (2021). Performance Analyses of a RAIM Algorithm for Kalman Filter with GPS and NavIC Constellations. Sensors.

[22] Sun Y. (2021). Autonomous Integrity Monitoring for Relative Navigation of Multiple Unmanned Aerial Vehicles. Remote Sens..

[23] Li Q., Wang D., Wu J., Wang L. (2025). New approach for integrity risk evaluation based on expected acceptable region for carrier-phase-based precise relative positioning. GPS Solut..

[24] Tang S., Li H., Pau C. Single Difference Code-Based Technique for Direct Position Estimation. Proceedings of the Proceedings of the 37th International Technical Meeting of the Satellite Division of The Institute of Navigation.

[25] Blanch J., Walter T. (2021). Fast Protection Levels for Fault Detection with an Application to Advanced RAIM. IEEE Trans. Aerosp. Electron. Syst..

[26] Milner C., Pervan B. (2020). Bounding Fault Probabilities for Advanced RAIM. IEEE Trans. Aerosp. Electron. Syst..

[27] Shanghai ComNav Technology Ltd. (2018). K508 Multi-GNSS OEM Board Product Manual.

[28] (2021). BeiDou Navigation Satellite System Open Service Performance Standard (Version 3.0). http://www.beidou.gov.cn/xt/gfxz/.

